# Membrane-enriched proteome changes and prion protein expression during neural differentiation and in neuroblastoma cells

**DOI:** 10.1186/s12864-017-3694-6

**Published:** 2017-04-22

**Authors:** J. A. Macedo, D. Schrama, I. Duarte, E. Tavares, J. Renaut, M. E. Futschik, P. M. Rodrigues, E. P. Melo

**Affiliations:** 1 CBMR, Center for Biomedical Research, University of Algarve, Campus de Gambelas, Faro, Portugal; 2 CCMAR, Centre of Marine Sciences of Algarve, University of Algarve, Campus de Gambelas, Faro, Portugal; 3 LIST, Luxembourg Institute of Science and Technology, Belvaux, Luxembourg; 4 School of Biomedical & Healthcare Sciences, Plymouth University Peninsula Schools of Medicine and Dentistry, Plymouth, UK

**Keywords:** Prion protein, 2D-DIGE, Neural differentiation, Chaperone activity, Redox homeostasis

## Abstract

**Background:**

The function of the prion protein, involved in the so-called prion diseases, remains a subject of intense debate and the possibility that it works as a pleiotropic protein through the interaction with multiple membrane proteins is somehow supported by recent reports. Therefore, the use of proteomic and bioinformatics combined to uncover cellular processes occurring together with changes in the expression of the prion protein may provide further insight into the putative pleiotropic role of the prion protein.

**Results:**

This study assessed the membrane-enriched proteome changes accompanying alterations in the expression of the prion protein. A 2D-DIGE approach was applied to two cell lines after prefractionation towards the membrane protein subset: an embryonic stem cell line and the PK1 subline of neuroblastoma cells which efficiently propagates prion infection. Several proteins were differentially abundant with the increased expression of the prion protein during neural differentiation of embryonic stem cells and with the knockdown of the prion protein in PK1 cells. The identity of around 20% of the differentially abundant proteins was obtained by tandem MS. The catalytic subunit A of succinate dehydrogenase, a key enzyme for the aerobic energy metabolism and redox homeostasis, showed a similar abundance trend as the prion protein in both proteomic experiments. A gene ontology analysis revealed “myelin sheath”, “organelle membrane” and “focal adhesion” associated proteins as the main cellular components, and “protein folding” and “ATPase activity” as the biological processes enriched in the first set of differentially abundant proteins. The known interactome of these differentially abundant proteins was customized to reveal four interactors with the prion protein, including two heat shock proteins and a protein disulfide isomerase.

**Conclusions:**

Overall, our study shows that expression of the prion protein occurs concomitantly with changes in chaperone activity and cell-redox homeostasis, emphasizing the functional link between these cellular processes and the prion protein.

**Electronic supplementary material:**

The online version of this article (doi:10.1186/s12864-017-3694-6) contains supplementary material, which is available to authorized users.

## Background

Prion diseases, such as Creutzfeldt-Jakob disease (CJD) in humans, bovine spongiform encephalopathy (BSE) in cow and scrapie in sheep are fatal neurodegenerative protein misfolding diseases. In humans, the sporadic form of CJD accounts for the majority of cases [1]. Inherited prion disease occurs due to germline mutations in *PRNP* that predispose individuals to CJD, Gerstmann-Straussler-Scheinker Disease or Fatal Familial Insomnia. The acquired prion diseases include accidental inoculation during medical procedures (iatrogenic CJD) or exposure to food products contaminated with BSE (variant CJD) [2]. The prion protein (PrP) involved in these diseases is a conserved ubiquitously expressed glycoprotein most abundant in the central nervous system. The mature form is anchored to the cell membrane by a glycosylphosphatidylinositol (GPI) group. It has an alpha helix-rich C-terminal globular domain, containing two asparagine-linked glycosylation sites, an intramolecular disulphide bond, a hydrophobic central region and an unstructured N-terminal domain, containing five repeats of a copper-binding octapeptide [3]. The disease associated isoform, or scrapie prion protein (PrP^Sc^ to distinguish from the cellular form PrP^C^), has higher beta sheet content, propensity to aggregate and it is able to replicate by binding to cellular prion protein and refolding it into the scrapie conformation [2, 4].

The first results obtained with two distinct PrP null mouse strains suggested that either PrP is unnecessary for normal development or its absence is somehow compensated [5, 6]. Later constructs used to knockout PrP have shown a neurodegenerative phenotype, caused by ectopic expression of its homologue doppel [7–9]. However, the clearest phenotype of PrP knockout mice is resistance to prion infection and inability to replicate prions [10, 11]. Based on the mild phenotypic traits in these knockouts and on cell culture studies, PrP has been assigned roles in many biological processes including myelin maintenance, copper and zinc transport, calcium homeostasis, as well as neuroprotective activities against several toxic insults, such as oxidative and excitotoxic damage [11–13]. PrP was also shown to promote the self-renewal and to regulate the proliferation of haematopoietic stem cells, human embryonic stem (ES) cells and neural precursors [14–17]. Additionally, treatment of embryonic hippocampal neurons with recombinant PrP enhanced neurite outgrowth and survival [18]. Altogether, these reports suggest that PrP plays a role as a switch from uncommitted multipotent precursors towards the generation of neurons [19]. To confirm this, it was shown recently that silencing PrP suppressed differentiation of human ES cells towards ectodermal lineages indicating that expression of PrP guides differentiation into neuron-, oligodendrocyte-, and astrocyte-committed lineages [20].

Structurally, PrP does not span the membrane and cannot transduce signals into the cytosol, but due to its binding partners it has been proposed to be involved in the assembly of signalling complexes [4]. Accordingly, it is pivotal the identification of additional proteins involved in the cellular functions of PrP and, eventually, in the protein misfolding replicative mechanism that leads to infection. Therefore, this study focused on assessing the membrane-associated proteome changes occurring together with alterations in the expression of PrP, aiming at finding potentially new interacting proteins. Two cellular systems with opposite changes in the expression of PrP were used: in one the expression of PrP increased during neural differentiation of ES cells and, in the other one, we used a neuroblastoma cell line knockdown for PrP. The neuroblastoma cell line PK1 was selected for its ability to replicate PrP^Sc^ and for the availability of a counterpart PrP knockdown cell line [21]. The quantitative 2D-DIGE identified 25 differentially abundant proteins during neural differentiation of ES cells, most of them belonging to the heat shock protein (HSPs) and the protein disulfide isomerase (PDIs) families. For neuroblastoma PK1 cells knockdown for PrP (PK1-KD), 6 differentially abundant proteins were identified. In order to obtain further insight into the differentially abundant protein functions, a Gene Ontology (GO) analysis was undertaken, together with the comprehensive assembly of an individual interactome for each differentially abundant protein set. Interestingly, succinate dehydrogenase complex, subunit A, a key enzyme for the energy metabolism that catalyzes the oxidation of succinate to fumarate and that is essential to prevent oxidative stress [22], correlated with PrP levels in both experiments.

## Methods

### Cell lines and culture conditions

The conversion of murine ES cells into neuroepithelial precursors (NPs) in adherent monoculture was performed using the *Sox1*-GFP knock-in (46C) ES cell line [23]. ES cells were initially plated on gelatin-coated 60 mm dishes at a density of 3 × 10^4^ cells/cm^2^ in GMEM (Invitrogen), supplemented with 10% fetal bovine serum (ESC qualified FBS, Invitrogen), 2 ng/ml of leukemia inhibitory factor (produced and purified according to Mereau et al. [24]), 1× non-essential aminoacids (Invitrogen), 2 mM glutamine (Invitrogen), 1% penicillin/streptomycin (Invitrogen) and 1 mM 2-mercaptoethanol (Sigma). For monoculture differentiation, ES cells were dissociated with 0.5% trypsin and plated onto polylysine/laminin (Sigma) coated 6-well plates at a density of 2 × 10^4^ cells/cm^2^ in serum-free RHB-A medium (StemCell Science Inc.), supplemented with 5 ng/ml of FGF2 (Peprotech). Medium was renewed every two days. In an attempt to obtain a more uniform NPs population, a transient selection for 48 h with 0.5 mg/ml puromycin (the *Sox1*-GFP reporter is linked to a puromycin resistance gene by an internal ribosome entry site [23]) was performed after three passages, using accutase for gentle dissociation [25]. Fixed cells were imaged with an Axio Imager Z2 ApoTome microscope (Carl Zeiss). The mouse PK1 cells used herein are a subline of neuroblastoma N2a cells that efficiently propagates RML (Rocky Mountain Laboratory) prions [26]. PrP knockdown cells derived from PK1 cells [21] were also used in this study.

### RNA extraction and quantitative real-time PCR

Total RNA was extracted using the Quick-RNA MiniPrep (Zymo Research), for up to 5 × 10^6^ cells, with in-column DNase I treatment (Promega), according to the manufacturer’s protocol. Nucleic acid quantification was performed using a Nanodrop spectrophotometer (Thermo Fisher Scientific) and 1 μg of RNA was used to synthesize first-strand cDNA with qScript cDNA SuperMix (Quanta Biosciences), according to the manufacturer’s instructions. The cDNA was diluted 10-fold and 2 μl were used in each gene-specific PCR reaction, performed in triplicate. Quantitative real-time PCR (qPCR) was carried out on a CFX96 detection system (Bio-Rad) using SYBR Select Master Mix from CFX (Invitrogen). The qPCR was performed using specific primer pairs for the genes of interest (Additional file 1: Table S1), according to the following conditions: initial step at 95 °C for 5 min followed by 40 cycles at 95 °C for 1 s and 60 °C or 65 °C for 35 s. Changes in expression of the target genes were normalized to glyceraldehyde 3-phosphate dehydrogenase (*Gapdh*). Triplicate samples of cells were collected at each time point and qPCR was performed on the cDNA synthetized from three separate RNA preparations. For each of the three biological replicates, C_T_ values of three technical replicates were averaged and whenever CV (%) >1 the values were tested with Grubbs test (graphpad.com/quickcalcs/Grubbs1.cfm) for outlier detection. Data were analysed using the ΔΔC_T_ method (C_T_, Target – C_T_, *Gapdh*)_Time X_ – (C_T_, Target – C_T_, *Gapdh*)_Time 0_. Time X corresponds to the days of cell differentiation and Time 0 to the undifferentiated ES cells. The fold changes based on the 2^-ΔΔCT^ calculation [27] were obtained from the ΔΔC_T_ averaged means of the biological replicates.

### Flow cytometry

Cells were collected mechanically using cell scrapers, centrifuged at 1200 rpm for 5 min and resuspended in 4% FBS in PBS. Acquisition was performed in a FACSCalibur cytometer (Becton Dickinson). Live cells were gated based on forward and side scatters and GFP fluorescence presented as stacked histograms.

### Protein prefractionation with Triton X-114

Cells were resuspended in ice-cold PBS and then extracted by addition of 2% (v/v) Triton X-114 (Sigma-Aldrich) followed by 15 min on ice. The mixture was clarified by centrifugation, at 10000 g for 10 min at 4 °C, the supernatant transferred to a new tube and warmed at 37 °C until it became cloudy. The solution was then centrifuged at 1500 g for 10 min at room temperature (RT) to separate into two protein-containing phases due to aggregation of detergent micelles [28]. The lower phase enriched in detergent containing membrane associated proteins with an amphiphilic nature was precipitated with acetone for a minimum of 2 h at -20 °C. After centrifugation at 10000 g for 10 min at 4 °C, pellets were allowed to dry and then resuspended in CHAPS buffer. The protein samples were quantified by a Bradford microplate assay (BioRad).

### Western blotting

Protein samples (25 μg) were denatured with Laemmli buffer and heated at 95 °C for 10 min. The separation was performed by SDS-PAGE in 12.5% gels. Proteins were then transferred onto low fluorescence PVDF membranes (Thermo Fisher Scientific) by electroblotting at 300 mA for 1 h. After blocking overnight in 5% milk/TBS/0.05% Tween-20 (TBST), membranes were washed and probed with primary monoclonal antibody (POM1, Prionics; GRP78/HSP5, Thermo Fisher Scientific) in 1% milk/TBST for 1 h at RT. After three washes with TBST, membranes were probed with secondary antibody goat anti-mouse IgG conjugated to AF488 (Invitrogen). Subsequently, the membranes were thoroughly washed with TBST and allowed to dry before fluorescence imaging using a Typhoon Trio scanner (GE Healthcare). The membranes were reprobed with anti β-actin antibody for protein load verification, either directly conjugated to AF647 (Santa Cruz) or indirectly using a goat anti-mouse IgG conjugated to AF647 (Invitrogen).

### Two-dimensional differential gel electrophoresis

Two-dimensional differential gel electrophoresis (2D-DIGE) was performed in a mixture of protein samples, labelled according to the CyDye minimal labelling method (GE Healthcare). Cy2 was used for the internal standard, composed of a balanced amount of each of the samples, whereas Cy3 and Cy5 were used to label the different samples, applying a dye swap to avoid biasing of the results due to differential labelling. Labelled samples were pooled by three such that each pool contained an equal ratio of proteins marked with Cy2, Cy3 and Cy5. Finally, 5.4 μL of Destreak Reagent (GE Healthcare), 1% (v/v) of IPG buffer pH 3 − 11NL (GE Healthcare) and CHAPS lysis buffer were added to reach a final volume of 450 μl. Additionally to the analytical gels, preparative gels with a total protein load between 400-500 μg were run for Coomassie blue staining dedicated to spot picking.

The migration of the first dimension or isoelectric focusing (IEF) was carried out using IPG strips pH 3 − 11NL, 24 cm (GE Healthcare), after overnight passive rehydration. The IEF was carried out on an Ettan IPGphor 3 IEF unit (GE Healthcare) with the following parameters: (1) gradient to 250 V for 1 h, (2) constant voltage of 250 V for 1 h, (3) gradient from 250 V to 1000 V for 2 h, (4) gradient from 1000 V to 8000 V for 3 h and (5) constant voltage of 8000 V for 5 h 40 min. The temperature was set at 20 °C and the current was limited to 75 μA/strip. After the first dimension, strips were equilibrated in equilibration buffer containing 75 mM Tris-HCl pH 8.8, 6 M urea, 30% (v/v) glycerol, 2% (w/v) SDS complemented with 1% (w/v) DTT (AppliChem) for 15 min and subsequently 15 min in equilibration buffer complemented with 2.5% (w/v) iodoacetamide (GE Healthcare). Strips were rinsed in cathode buffer, placed on top of the second dimension gels and sealed with low-melt agarose (AppliChem). Cathode and anode buffers were added in the electrophoresis tank (Ettan DALT six, GE Healthcare) and the gels were run at 20 °C. The migration settings were: (1) 10 mA/strip for 1 h and (2) 40 mA/strip until the sample reached the end of the gel. The preparative gels dedicated to picking were fixed in 50% (v/v) ethanol containing 2% (v/v) phosphoric acid for a minimum of 2 h. Subsequently, gels were washed three times for 20 min with ddH_2_O and left overnight in an equilibration solution for coloration with 34% (v/v) methanol, 17% (w/v) aluminium sulphate, 2% (v/v) phosphoric acid and 3% (w/v) of Coomassie brilliant blue G250 (Amresco).

Gels were scanned using a Typhoon Trio Variable Mode Imager (GE Healthcare) at a resolution of 100 μm. The gel images were analysed using the software SameSpots (TotalLab). Spot comparisons between the samples were carried out by calculating the ratio between the average intensity of the conditions. When the average intensity of a spot measured in a differentiated sample exceeded the one measured in the ES sample, we reported a protein fold change equal to the ratio r. In the case where the spot intensity was lower than the one measured in ES cells (when r < 1), we reported a fold change equal to −1/r. Only significant absolute fold changes superior to 1.5 for the first experiment or 2.0 for the second experiment (ANOVA, *p*-value ≤ 0.05) were considered. Differentially abundant spots were matched onto the high protein load gels and picked from the latter for MS analysis.

### Mass spectrometry analysis and protein identification

Digestion and MALDI spotting were carried out using an Ettan spot handling workstation (GE Healthcare) or a Janus liquid handling workstation (Perkin Elmer), in the first and second experiment, respectively. In-gel tryptic digestion protocols were the standard used in each proteomics platform (CRP-Gabriel Lippmann, Luxembourg and GIGA, Belgium). The MALDI peptide mass spectra were acquired using either an AB SCIEX TOF/TOF 5800 (Applied Biosystems) or an UltrafleXtreme TOF/TOF (Bruker). All spectra, MS and MS/MS, were submitted for database dependent identification on the MASCOT server using either NCBInr (143978 sequences) or Swissprot with restricted taxonomy *Mus musculus*. The parameters used for these searches were mass tolerance MS 100 ppm, mass tolerance MS/MS 0.5 Da, fixed modifications cysteine carbamidomethylation and variable modifications methionine oxidation, double oxidation of tryptophan and tryptophan to kynurenine. Proteins were considered identified when at least two peptides passed the MASCOT-calculated threshold score (*p* < 0.05).

### Bioinformatics analysis

The molecular interaction network for each set of proteins differentially abundant in our analyses was obtained by generating a comprehensive list of physical interactions gathered from five online databases: StemCellNet [29], UniHI [30], STRING [31], GeneMANIA [32] and QIAGEN’s Ingenuity Pathway Analysis [33]. The data retrieved from these sources were formatted, filtered and merged using customized Bash and R scripts. To build a high-confidence interaction data set, we selected only experimental data (i.e. excluding computationally predicted interactions) and direct interactions with our proteins of interest, generating a final list containing 1993 physical interactions for the first set of differentially abundant proteins (Additional file 2: Figure S1). Building this customized interaction data set was necessary to overcome the minimal agreement between different databases (Additional file 3: Figure S2). The network connecting the differentially abundant proteins was generated using Cytoscape version 3.3.0 [34]. A GO enrichment analysis, using *Mus musculus* as reference, was performed on both data sets for cellular component using PANTHER [35] and the overrepresented categories considered significant at *p* < 0.05 after Bonferroni correction. Additionally, a GO enrichment analysis was conducted for the full interactome of the first data set, using Cytoscape plugin BiNGO version 3.0.3 [36]. BiNGO analyses were performed using custom updated GO and mouse GO annotation files (downloaded from the Gene Ontology Consortium website), discarding the GO evidence codes ISS, IEA, NAS and ND. The overrepresentation was considered significant at *p* < 0.05 after Bonferroni correction.

## Results

### Pluripotency and neural differentiation markers expression in differentiating ES cells

**Fig. 1:**
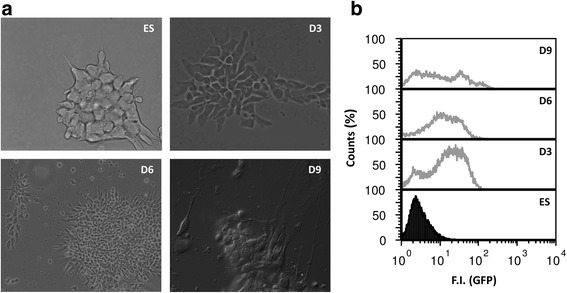
**a**) Phase contrast images during ES cells differentiation. For day 9 a differential interference contrast (DIC) image was chosen to highlight axonal projections being formed particularly in the periphery of rosettes. **b**) Flow cytometry profile of *Sox1-GFP* activation for the same time points of ES cells differentiation

**Fig. 2:**
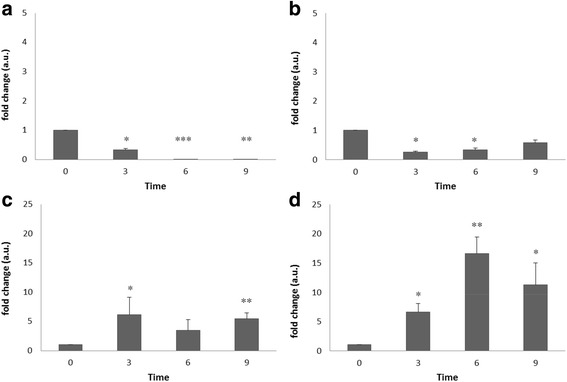
**a**-**d**) Quantitative mRNA expression of different markers during ES cells differentiation (0, 3, 6, 9 days). a) *Oct4*, b) *Nanog*, **c**) *Sox1* and **d**) *Nestin*. Pairwise t-tests were performed to calculate significant differences compared to ES cells (*p < 0.05, **p < 0.01, ***p < 0.001)

Neural differentiation of the ES cell line 46C in adherent monoculture was followed morphologically and through expression of *Sox1-GFP* knock-in reporter (Fig. 1). Sox1 is the earliest known specific marker of neuroectoderm in the mouse embryo [23]. As previously reported, undifferentiated 46C ES cells do not express *Sox1-GFP* and an increase in GFP fluorescence is observed during differentiation to NPs [37]. These values tend to be more variable on day 3, due to differences on the onset of differentiation, and reach a plateau from day 4 to day 6. Flow cytometry analysis during ES cells differentiation to NPs showed some heterogeneity in *Sox1-GFP* positive cells that decline after day 6. The full transcriptome of neural differentiation of 46C ES cells was previously characterized [37] and the culture conditions were mimicked in our study, without splitting to avoid interfering with PrP expression on the membrane. The qPCR results confirm neural differentiation of ES cells showing significant downregulation of the stemness markers *Oct4* and *Nanog* during the process of neural conversion (Fig. 2). *Sox-1* levels increased on day 3 and lowered from day 3 to days 6 and 9 corresponding approximately to the flow cytometry data, although high statistical significance was only attended at the latter time point due to lower variability. *Nestin* widely used as a neural stem/progenitor cell marker increased its expression with statistical significance (Fig. 2 a-d).

### Prion protein expression during the differentiation of ES cells into NPs

**Fig. 3:**
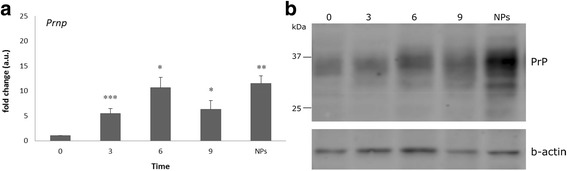
**a**) Prion protein mRNA expression during ES cell differentiation. Pairwise t-tests were performed to calculate significant differences compared to ES cells (*p < 0.05, **p < 0.01, ***p < 0.001). **b**) Western blot of PrP expression during ES cell differentiation; β–actin was used as loading control

The expression of PrP was evaluated both at the mRNA level, by qPCR, and at the protein level, by western blot (Fig. 3a and 3b). Levels of mRNA increased upon trigger of differentiation with some large variation on days 6 and 9. The highest consistent levels were found at the NPs stage. The expression of PrP was analysed by western blot after Triton X-114 prefractionation. The analysis showed bands between 25 and 37 kD, corresponding to differentially glycosylated isoforms (unglycosylated, monoglycosylated and its most abundant diglycosylated form). The levels of PrP showed an increasing trend along the time of differentiation, reaching its highest in NPs as observed at mRNA. These results confirm that PrP levels are directly associated with neuronal differentiation, hence playing a role both in neurogenesis and in cellular differentiation [19].

### Proteins differentially abundant during neural differentiation of ES cells

**Fig. 4:**
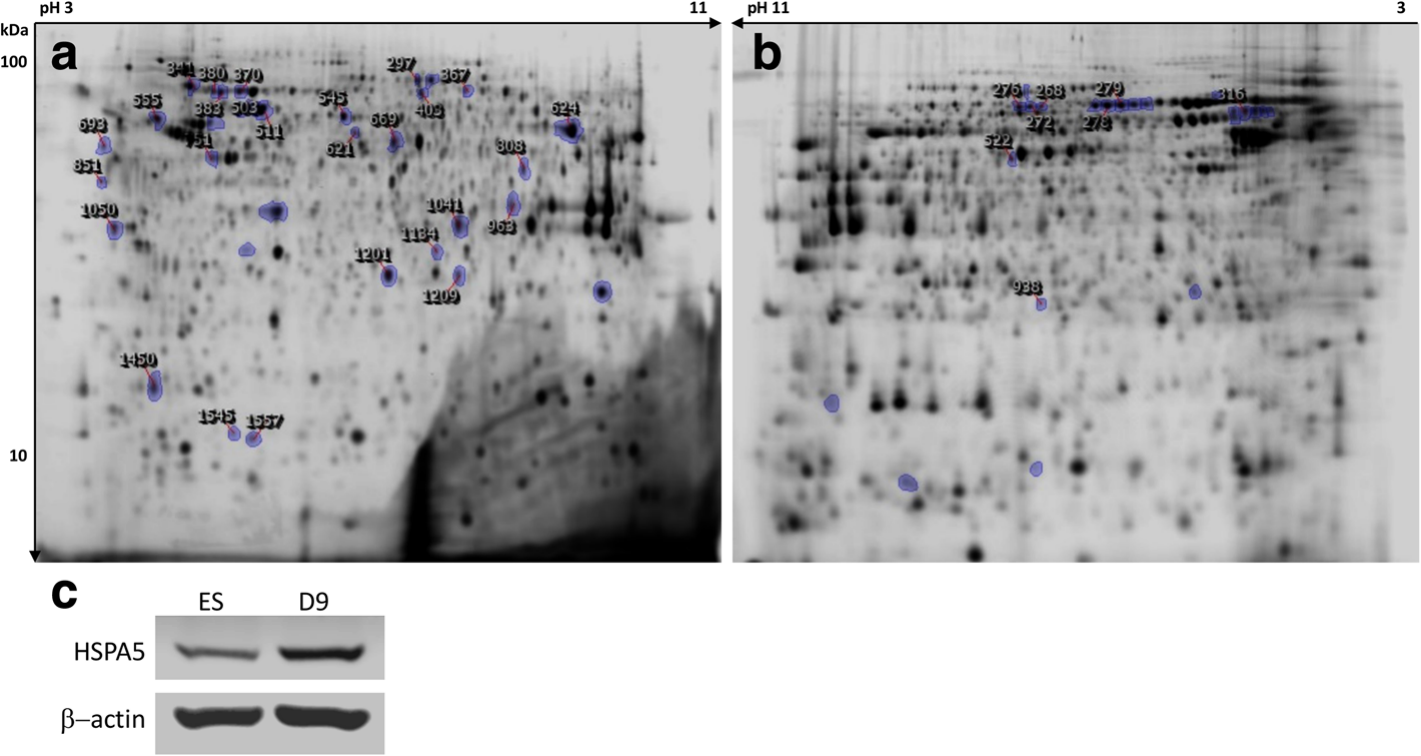
**a**) 2D-DIGE Cy2 reference gel comparing ES differentiating cells. **b**) 2D-DIGE Cy2 reference gel comparing PK1 and PK1-KD cells. Significant differences were calculated by ANOVA (*p* < 0.05) and *marked spots* correspond to picked spots with ± 1.5 or 2.0 fold changes for **a**) and **b**), respectively. *Numbered spots* correspond to the identified proteins. **c**) Western blot for detection of HSPA5 in ES compared to day 9 cells

Triton X-114 prefractionation was demonstrated to enable the selective enrichment of hydrophobic proteins [28] and followed by 2D-PAGE remains a method of choice for proteomic characterization of the membrane protein subset [38]. In our study, we have decided for this simple enrichment step that rendered the protein yield suitable for quantitative 2D-DIGE. Subsequently, the GO enrichment analysis for cellular component validated the use of this prefractionation step to enrich for membrane-associated proteins. ES cells were allowed to differentiate for 9 days and samples were collected from three time points: ES cells, D6 and D9. The time point D3 was excluded from sampling due to possible differences on the onset of differentiation. Three conditions with four biological replicates were selected for a single run. The gel analysis, after manual validation of the spots with *p* < 0.05 (ANOVA), revealed 158 spots with significantly different abundance (Fig. 4a). From these, we confidently picked 32 of which 29 were successfully identified as corresponding to 25 distinct proteins. These proteins are shown in Table 1 with indication of the fold change during the differentiation process, associated gene nomenclature, theoretical molecular weight, pI and sequence coverage by tandem MS. A more complete table, including the accession number ID and Mascot scores is available (Additional file 1: Table S2). From the 25 distinct proteins identified as differentially abundant, 14 proteins were shown to increase and 11 to decrease its levels during the differentiation of ES cells. The protein identified as cellular retinoic acid-binding protein I (CRABPI) had the highest increase of 13.7 fold change and heat shock protein beta-1 (HSPB1) had the highest decrease of 11.0 fold change. Figure 4c shows a western blot corroborating the 2D-DIGE increase in HSPA5, one of PrP direct interactors [39] that highlights the importance of protein folding as the biological process most significantly enriched concomitantly with changes in PrP expression (see discussion). Global gene expression profiling using Affymetrix microarrays for the same cell line was previously published [37] and we compared our results to their report using the same cut-off parameter of ± 1.5 fold change (Table 1). From the 14 proteins for which the comparison was possible, 12 proteins were in agreement with the previous results. The two exceptions showing contrary fold changes between protein and mRNA levels were the mitochondrial inner membrane protein (IMMT) and the phosphoglycerate mutase 1 (PGAM1).

**Table 1:** Proteins differentially abundant during neural differentiation of ES cells. Fold change refers to increased levels (positive FC) or decreased levels (negative FC) of protein content from ES to D6 and D9. Fold change ± 1.5 (ANOVA *p* < 0.05) was used as threshold for protein identification

Spot #	Protein [*Mus musculus*]	Gene	Mw(kDa)	pI	SC(%)	FC	Array*
297	Inner membrane protein, mitochondrial	*Immt*	80.9	6.8	62	+1.8	↓^b^
341	78 kDa glucose-regulated protein precursor	*Hspa5*	72.5	5.1	45	+1.6	--
367	Far upstream element-binding protein 1	*Fubp1*	67.3	7.2	61	+2.1	↑^a^
370	Heat shock cognate 71 kDa protein	*Hspa8*	42.4	6.7	62	−1.6	↓^a^
380; 383	Lamin-B1^c^	*Lmnb1*	67.0	5.1	56	+2.4; +2.8	--
403	Succinate dehydrogenase [ubiquinone] flavoprotein subunit, mitochondrial	*Sdha*	73.6	7.1	53	+2.1	--
503; 511	60 kDa heat shock protein, mitochondrial^c^	*Hspd1*	59.6	8.1	44	−1.8; − 2.0	--
545	Protein disulfide-isomerase A3	*Pdia3*	57.1	5.9	49	+1.9	--
555	Protein disulfide-isomerase	*P4hb/Pdia1*	57.4	4.8	45	+1.6	--
621	Heterogeneous nuclear ribonucleoprotein H	*Hnrph1*	47.9	6.1	46	+1.9	↑^a^
624	ATP synthase, mitochondrial F1 complex, alpha subunit	*Atp5a1*	54.9	9.4	14	+1.9	--
669	Enolase 1B	*Eno1*	47.5	6.4	62	−1.8	↓^a^
693	Reticulocalbin-2	*Rcn2*	37.3	4.3	38	+2.4	↑^a^
751	Actin, cytoplasmic 1	*Actb*	42.1	5.3	45	−1.7	--
808	Mitochondrial import receptor subunit TOM40	*Tomm40*	38.3	7.6	58	−1.9	↓^a^
851	mCG49244	*C9orf156*	21.8	5.7	76	+2.6	--
963	Glyceraldehyde-3-phosphate dehydrogenase	*Gapdh*	36.1	8.4	15	−2.0	--
1041	Voltage-dependent anion-selective channel protein 2	*Vdac2*	32.3	7.4	65	−1.6	↓^a^
1050	YL2 protein	*C1qbp*	23.8	4.4	13	−2.6	↓^a^
1134	Phosphoglycerate mutase 1	*Pgam1*	28.9	6.7	77	+1.8	↓^b^
1201	Heat shock protein beta-1	*Hspb1*	23.1	6.1	26	−11.0	↓^a^
1209	Glutathione S-transferase A4	*Gsta4*	25.6	6.8	49	−3.4	↓^a^
1450	Cytochrome b-5, type B	*Cyb5b*	13.3	5.7	41	−1.9	--
1545	Fatty acid binding protein 7, brain	*Fabp7*	15.2	5.1	87	+7.6	↑^a^
1557	Cellular retinoic acid-binding protein I	*CrabpI*	15.6	5.3	92	+13.7	↑^a^

*SC* sequence coverage, *FC* fold change

*Microarray trend in expression comparing ES to D8 is shown for the available protein-coding genes [37]; data from probe sets giving conflicting results for the same gene and fold changes below the cut-off of 1.5 were excluded. ^a^same trend; ^b^opposite trend; ^c^proteins identified in two different spots

### Proteins differentially abundant in neuroblastoma cells expressing the prion protein and knockdown for the prion protein

Quantitative 2D-DIGE was also performed for comparison between PK1 neuroblastoma cells expressing PrP and PK1-KD which are knockdown for PrP, aiming at gathering information about the effect of PrP ablation on the membrane enriched proteome. The levels of PrP in PK1-KD cells are almost undetectable by western blot and immunocytochemistry [21, 40]. From a selection of 41 spots with significantly different abundance, 23 were picked resulting in the identification of 8 spots and 6 distinct proteins (Fig. 4b and Table 2). From the 6 distinct proteins identified, 4 were higher and 2 lower in PK1-KD when compared to PK1 cells. Comparing the two sets of identified proteins in ES cells and PK1 cells, succinate dehydrogenase complex flavoprotein subunit A (SDHA) was detected as common protein, whilst the alpha subunit of mitochondrial ATP synthase (ATP5A1) was identified in the first set and the beta subunit (ATP5B) was identified in the second set. Interestingly, SDHA increased during neural differentiation concomitantly with increased levels of PrP and was found to be lower in cells not expressing the latter. For PK1 cells, SDHA was identified in three different spots that were decreased with similar fold change in PK1-KD cells (-2.5 ± 0.5). ATP5A1 increased upon neural differentiation, concomitantly with the levels of PrP, but ATP5B also increased significantly in PK1-KD cells. PK1 cells, which are highly efficient in the replication of RML prions [26], were infected as previously described [40] and a 2D-DIGE experiment comparing non-infected and infected cells was carried out. This experiment gave no significant differential abundance of proteins (data not shown), which was not surprising since no transcriptional changes seem to be induced by prion infection of neural cell lines [41]. Transcriptome changes seem to occur between susceptible and resistant PK1 subclones [42] but were not induced by infection [41].

**Table 2:** Proteins differentially abundant in PK1 neuroblastoma cells knockdown for the prion protein. Four proteins showed increased levels (positive FC) and two decreased levels (negative FC) in knockdown cells. Fold change ± 2 (ANOVA *p* < 0.05) was used as threshold for protein identification

Spot #	Protein [*Mus musculus*]	Gene	Mw(kDa)	pI	SC(%)	FC
268; 272; 276	Succinate dehydrogenase [ubiquinone] flavoprotein subunit, mitochondrial^c^	*Sdha*	73.6	7.3	16	−2.1; − 2.3; − 3.0
278	Structural maintenance of chromosomes protein 2	*Smc2*	13.5	9.1	19	+4.4
279	T-complex protein 1 subunit theta	*Cct8*	60.1	5.3	31	+4.7
316	ATP synthase subunit beta, mitochondrial	*Atpb*	56.3	5.1	17	+6.3
522	Elongation factor Tu, mitochondrial	*Tufm*	49.9	7.9	14	−2.3
938	Acyl-protein thioesterase 1	*Lypla1*	25.0	6.2	10	+2.0

*SC* sequence coverage, *FC* fold change

^c^SDHA identified in three different spots with similar fold differences

### GO enrichment analyses and interactome of differentially abundant proteins

**Fig. 5:**
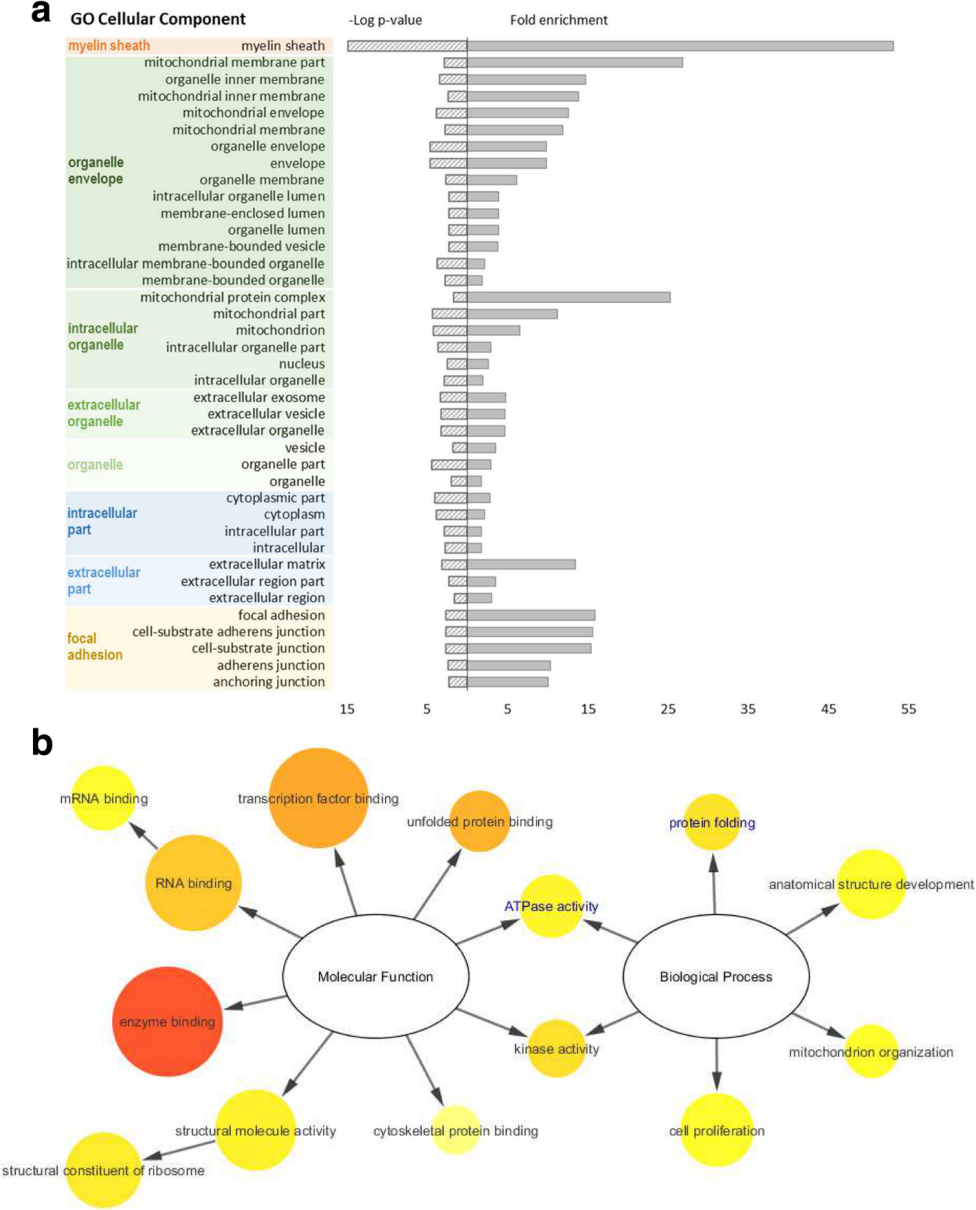
**a**) GO enrichment for cellular component, according to PANTHER analysis, for the differentially abundant proteins during neural differentiation of ES cells. The results are represented as –log *p*-value (*left axis*) and fold enrichment (*right axis*) for each category using *Mus musculus* as reference. The GO terms were clustered based on similarity. **b**) GO slim enrichment terms related to molecular function and biological process categories, calculated with BiNGO on Cytoscape, using the full interactome data set. Circle size is proportional to the frequency of the GO term, while colour indicates the –log *p*-value (*orange* for *higher*, *yellow* for *lower*). GO terms in *blue* represent the ones also found significant when testing only the differentially abundant proteins

**Fig. 6:**
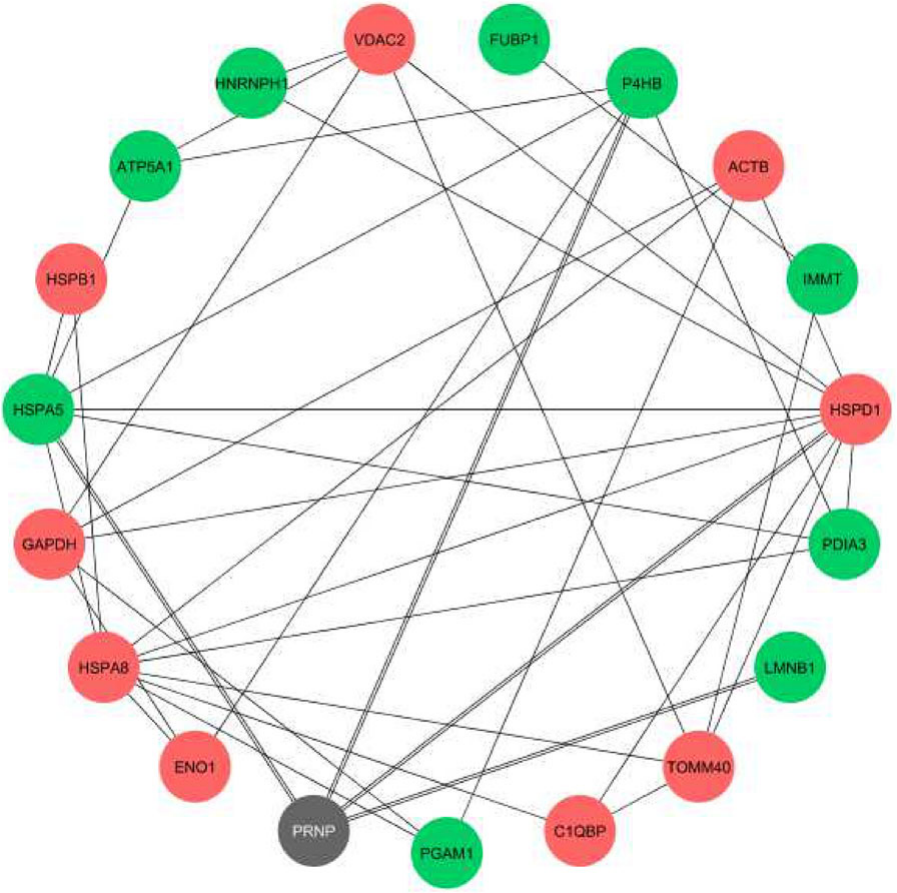
Physical interactions between the differentially abundant proteins during differentiation of ES cells. Proteins with increased expression (*n* = 9) are represented by *green* nodes and with decreased expression (*n* = 9) by *red* nodes, while PrP (here designated with the official queried symbol PRNP) is shown in *grey*. Orphan protein nodes (*n* = 7) are not shown

To validate the prefractionation technique and its ability to successfully enrich the sample with membrane-associated proteins, we conducted a GO enrichment analysis for cellular component. Figure 5a shows the results for the first experimental set. This analysis revealed that the identified proteins were mainly associated with the membrane of intracellular organelles, namely the mitochondria. “Focal adhesion” was also significantly enriched in the first set regarding neural commitment. Interestingly, both sets of identified proteins were enriched in “myelin sheath” associated proteins (*n* = 12, *p* < 0.001 in the first set and *n* = 3, *p* < 0.02 in the second set). The sets of differentially abundant proteins were too small to show a representative enrichment in GO molecular function or GO biological process categories. Accordingly, we proceeded with generating a customized interactome for the 25 differentially abundant proteins during differentiation, plus PrP, selecting only physical interactions which have been experimentally corroborated (Additional file 2: Figure S1). This approach aimed at gaining better insight into the relevant functions and biological processes present in the full interactome of our identified proteins (Fig. 5b). The most represented and prominent molecular functions found were “enzyme binding” and “transcription factor binding”. “Anatomical structure development” and “cell proliferation” were the most represented biological processes while “protein folding” and “kinase activity” were more significantly enriched (*vide p*-values). For the set of differentially abundant proteins alone, both “protein folding” and “ATPase activity” were also significantly enriched (Fig. 5b, GO terms in blue). According to our analysis, the physical interactions between the proteins differentially abundant upon neural commitment, including PrP, are shown in Fig. 6. From the 25 identified, 18 proteins had at least one interactor whilst 7 of them were orphan nodes. The main interactors were HSPs, namely HSPD1 (*n* = 10), HSPA8 (*n* = 9) and HSPA5 (*n* = 7). For PrP, four known interactors were identified: HSPD1, HSPA5, P4HB/PDIA1 and LMNB1 [39, 43, 44]. Other PrP interactors not reported in the resorted databases are PDIA3, ATP5A1, GAPDH and β-actin [45–48]. For the second set of differentially abundant proteins between PK1 and PK1-KD cells (Table 2), no interactors with PrP were found and a single experimental interaction between ATP5B and acyl-protein thioesterase 1 (LYPLA1) was reported in all queried databases.

## Discussion

Several functions have been assigned to PrP spanning from neurotransmission, olfaction, proliferation and differentiation of neural precursors, myelin maintenance, copper and zinc transport, calcium homeostasis as well as neuroprotective activities against several toxic insults such as oxidative and excitotoxic damage [13]. How does this protein gather so many functions? Does the answer lie in its ability to function as a pleiotropic protein through the interaction with multiple different membrane proteins? Two cellular systems, combined with a quantitative membrane-enriched proteomic analysis, were used in this study to identify proteins possibly related to the function of PrP. Firstly, ES cells were induced to differentiate into NPs as PrP has been proposed to participate in transmembrane signalling processes associated with neuronal differentiation during early embryogenesis and in adult neurogenesis [19, 49]. Recently, it was shown that silencing PrP suppressed differentiation of human ES cells towards ectodermal lineages indicating that expression of PrP guides differentiation rather than resulting from differentiation [20]. Indeed, levels of PrP increased during the differentiation of ES cells and were higher at the latest stage analysed (Fig. 3). Secondly, the PK1 neuroblastoma cell line that efficiently propagates RML prions [26] and PK1-KD, its knockdown counterpart for PrP [21], were analysed for membrane-enriched proteome changes. From the set of differentially abundant proteins upon neural differentiation, 25 proteins were identified (fold change ≥ ± 1.5) where 14 were shown to increase and 11 to decrease its expression levels (Table 1). The highest fold differences were the increases in proteins belonging to the intracellular lipid binding proteins superfamily, FABP7 and CRABPI, involved in binding intracellular hydrophobic ligands and trafficking them throughout cellular compartments [50]. Knockdown of PrP has resulted in the identification of 6 distinct proteins differentially abundant (fold change ≥ ± 2), where 4 showed higher and 2 lower expression levels (Table 2).

The quantitative results obtained with 2D-DIGE were further explored using bioinformatics tools to build protein networks and gain indications about possible pathways involved in neural differentiation and in the role of PrP. GO enrichment analysis for the cellular component category carried out for the 25 differentially abundant proteins upon neural commitment revealed an enrichment for “myelin sheath”, “organelle membrane” and “focal adhesion” associated proteins, reflecting not only the neural features of differentiation but also the prefractionation step used to select the membrane protein subset (Fig. 5a). Enrichment in proteins involved in “focal adhesion” might be consistent with a role of PrP in the modulation of cell adhesion and stability of adherens cell junctions during embryonic development [51]. The enrichment analysis performed for the GO molecular function and GO biological process was done for the customized interactome, built for the 25 differentially abundant proteins in order to strengthen the functional enrichment analysis. “Enzyme binding” appeared as the most prominent molecular function and “protein folding” and “kinase activity” as the most significantly enriched biological processes associated to the neural differentiation of ES cells (Fig. 5b). Interestingly, using the set of differentially abundant proteins alone, “protein folding” and “ATPase activity” were also significantly enriched biological processes. A previous proteomic study [52] also reported "protein folding" as the biological process associated to changes in the expression of PrP. Moreover, a recent shotgun proteomic study using CRISPR-Cas9-based knockout of PrP in NMuMG cells found that “cell adhesion”, “epithelial cell differentiation” and “response to inorganic substances” were biological processes significantly enriched for proteins with altered levels, possibly reflecting the ability of this cell line to undergo epithelial-to-mesenchymal transitions [53]. The lack of overlap between the proteins identified in different proteomic studies could be attributed to cell type-specific effects of PrP pleiotropy, as previously reported [52, 53].

The interactome for the specific set of proteins differentially abundant during neural differentiation of ES cells was obtained to gather insight into putative functional links between PrP and other relevant proteins. Our interactome revealed four interactors with the PrP (HSPD1, HSPA5, P4BH/PDIA1 and LMNB1, see Fig. 6). Although, not reported in the resorted databases, PDIA3 was demonstrated to interact with PrP in N2a cells by co-immunoprecipitation [45], β-actin was found to interact with PrP in junctional domains of enterocytes [46] and two other members of the interactome (ATP5A1 and GAPDH) were found to co-purify with a myc tagged PrP from transgenic mouse brains [47]. From these possible interactors with PrP, six (HSPD1, HSPA5, PDIA3, ATP5A1, GAPDH and β-actin) are annotated as “myelin sheath” associated proteins. Several of the proteins co-purified with myc tagged PrP also belonged to the group of myelin-associated proteins [47], suggesting that enrichment in myelin-associated proteins revealed by our GO analysis might not be due merely to a neural signature of the cell lines. Some interactors might even be possible candidates for mediation of PrP myelinotrophic effects [54]. Additionally, our results showed that differentiation is accompanied with change in expression patterns of HSPs (Fig. 5b and Table 1). A subpopulation of HSPs was shown to associate with membranes and to play a role in membrane quality control [55]. It is emphasized that different HSPs have been found to associate, to a variable extent, with detergent-resistant microdomains (“rafts”) and this association can be modulated by stress. The membrane microdomain-associated HSPs can evidently participate in the orchestration and activity of distinct raft-associated signalling platforms [56–58]. We have identified four HSPs (HSPA5, HSPA8, HSPD1, HSPB1), mostly with decreased expression during the cells’ differentiation process, with the exception of the PrP interactor HSPA5. This chaperone, also known as 78 kDa glucose regulated protein (GRP78 or BiP), is the best studied chaperone to date with regard to protein folding disorders of the brain due to its central role in the activation of the unfolded protein response (UPR) [59]. Mice deficient in HSPA5, specifically in Purkinje cells, show activation of the UPR, apoptotic cell death and cerebellar atrophy, ultimately leading to death [60], emphasizing the importance of HSPA5 neuronal expression. In patients with sporadic CJD and in mice with scrapie, an increase in the levels of molecular chaperones such as GRP58, HSPA5 and HSP70 has been reported [61–63].

Two PDIs, PDIA1/P4HB and PDIA3 also known as GRP58, increased concomitantly with the expression of PrP upon neural differentiation and were previously reported to interact with the latter. Increased expression of PDIA1 following stable PrP overexpression in neuronal SH-SY5Y cells was reported in another proteomic study [52]. PDIA3 was also shown to be active in the plasma membrane, in which it is located in lipid rafts and binds to N-glycosylated proteins [64]. Both PDIs can act as chaperones and catalyze thiol/disulfide exchange, which may generate one hydrogen peroxide molecule per each disulfide bond formed in client proteins [65]. In line with this, PrP is an N-glycosylated protein with one intramolecular disulfide bridge mainly located in lipid rafts [66]. Recently, it was shown that PDIA3 is highly expressed in the brain of sporadic and infectious forms of prion diseases and controls the maturation and total levels of PrP [67].

SDHA and two different ATP synthase subunits were identified in both proteomic experiments performed, but only SDHA showed a similar abundance trend as PrP, i.e., increased abundance upon neural differentiation and decreased abundance in PK1-KD cells. SDHA is a key enzyme to attenuate the generation of reactive oxygen species associated with the aerobic energy metabolism in the mitochondria [22]. PDIA3, PDIA1 and SDHA are all players involved in the redox homeostasis of cells. The increased abundance observed for these three proteins plus PrP may well be related to an ubiquitous role of PrP in cell-redox homeostasis, where it may act as a sensor for oxidative stress [68–71]. PrP expression was previously shown to increase under oxidative stress [69, 72, 73] and neurodegeneration mediated by PrP is accompanied by a burst of reactive oxygen species and suppressed by antioxidants [68].

## Conclusions

Two cellular systems, where the expression of PrP changes very significantly, were used to identify proteins and GO categories possibly related to the function of PrP. Firstly, ES cells were induced to differentiate into NPs, process accompanied by a significant increase in the expression of PrP. Secondly, neuroblastoma PK1 cells were compared to its knockdown counterpart. From the set of differentially abundant proteins, the main following conclusions were extracted: (*i*) “protein folding” was the most significant enriched biological process occurring with changes in the expression of the prion protein in the first cell model used in this study, (*ii*) interactors with PrP identified in our study are annotated mostly as “myelin sheat” associated proteins and therefore may mediate a myelinotrophic effect of PrP [54], (*iii*) succinate dehydrogenase, a key enzyme to attenuate the generation of reactive oxygen species which is associated with the aerobic energy metabolism, showed a similar abundance trend as PrP in both cell systems and (*iv*) protein disulfide isomerases, a class of enzymes involved in cell-redox homeostasis [65, 74], previously reported as PrP interactors [45, 67], were also identified in our study as differentially abundant. Overall, our study shows that PrP expression occurs concomitantly with changes in chaperone activity and cell-redox homeostasis, emphasizing the functional link between these cellular processes and PrP.

## Additional files

Additional file 1: Table S1. and **Table S2.** Oligonucleotides used for qPCR of mouse specific genes & Protein spots differentially abundant during neural differentiation of ES cells (set #1, fold change ± 1.5) and between neuroblastoma cells expressing the prion protein and knockdown for the prion protein (set #2, fold change ± 2). (DOCX 22 kb)

Additional file 2: Figure S1. Hierarchical representation of the full interactome displaying all the physical interactions found on the queried databases between our first set of differentially abundant proteins, including prion protein (magenta diamonds) and their first interacting neighbours (green circles). (TIF 569 kb)

Additional file 3: Figure S2. Venn diagram displaying intersections as common number of physical interactions between the five databases used to retrieve the data for the bioinformatics analysis. (TIF 240 kb)

## Abbreviations

2D-DIGE: 2D-differential gel electrophoresis
BSE: Bovine Spongiform Encephalopathy
CJD: Creutzfeldt-Jakob disease
ES: Embryonic stem
GO: Gene Ontology
GPI: Glycosylphosphatidylinositol
HSP: Heat-shock protein
KD: Knockdown
NP: Neuroepithelial precursors
PDI: Protein disulfide isomerase
PrP: Prion protein
qPCR: Quantitative real-time PCR
RML: Rocky Mountain Laboratory
