# Supplementary material for: Membrane-enriched proteome changes and prion protein expression during neural differentiation and in neuroblastoma cells
Source: BMC Genomics. 2017 Apr 22;18:319. doi: 10.1186/s12864-017-3694-6 (PMC5401558; doi:10.1186/s12864-017-3694-6)
Supplement: Supplementary file 1 — and Table S2. Oligonucleotides used for qPCR of mouse specific genes & Protein spots differentially abundant during neural differentiation of ES cells (set #1, fold change ± 1.5) and between neuroblastoma cells expressing the prion protein and knockdown for the prion protein (set #2, fold change ± 2). (DOCX 22 kb) [file 12864_2017_3694_MOESM1_ESM.docx]

**Supplementary Material**

**Table S1.** Oligonucleotides used for qPCR of mouse specific genes.

|  | **Forward primer** | **Reverse primer** | **Size (bp)** |
| --- | --- | --- | --- |
| ***Gapdh*** | TCCCACTCTTCCACCTTCGATGC | GGGTCTGGGATGGAAATTGTCAGG | 247 |
| ***Oct4*** | GCAGGAGCACGAGTGGAAAGCAAC | CCAGGCCTCGAAGCGACAGATG | 270 |
| ***Nanog*** | ctcatcaatgcctgcagtttttca | ctcctcagggcccttgtcagc | 193 |
| ***Sox1*** | CATCTCCAACTCTCAGGGCT | ACTTGACCAGAGATCCGAGG | 226 |
| ***Nestin*** | GGAAGAAGTTCCCAGGCTTC | ATTAGGCAAGGGGGAAGAGA | 138 |
| ***Prnp*** | CAACCGAGCTGAAGCATTCTG | CGACATCAGTCCACATAGTC | 133 |

**Table S2.** Protein spots differentially abundant during neural differentiation of ES cells (set #1, fold change ±1.5) and between neuroblastoma cells expressing the prion protein and knockdown for the prion protein (set #2, fold change ±2).

|  | **Spot #** | **Accession ID** | **Protein designation** | **Gene** | **Mw (kDa)** | **pI** | **Score** | **SC(%)** | **FC** |
| --- | --- | --- | --- | --- | --- | --- | --- | --- | --- |
| **Set #1 (NCBInr)** | 297 | gi\|148666538 | Inner membrane protein, mitochondrial, isoform CRA_a | *Immt* | 80.9 | 6.8 | 1010 | 62 | +1.8 |
|  | 341 | gi\|254540166 | 78 kDa glucose-regulated protein precursor | *Hspa5* | 72.5 | 5.1 | 960 | 45 | +1.6 |
|  | 367 | gi\|568923199 | PREDICTED: far upstream element-binding protein 1 isoform X15 | *Fubp1* | 67.3 | 7.2 | 789 | 61 | +2.1 |
|  | 370 | gi\|215261181 | Chain A, chaperone complex | *Hspa8* | 42.4 | 6.7 | 879 | 62 | - 1.6 |
|  | 380; 383 | gi\|1882199589 | Lamin-B1 | *Lmnb1* | 67.0 | 5.1 | 829; 529 | 56; 48 | +2.4; +2.8 |
|  | 403 | gi\|54607098 | Succinate dehydrogenase [ubiquitone] flavoprotein subunit, mitochondrial precursor | *Sdha* | 73.6 | 7.1 | 976 | 53 | +2.1 |
|  | 503; 511 | gi\|76779273 | Hspd1 protein, partial | *Hspd1* | 59.6 | 8.1 | 140; 547 | 17; 44 | - 1.8; - 2.0 |
|  | 545 | gi\|112293264 | Protein disulfide-isomerase A3 precursor | *Pdia3* | 57.1 | 5.9 | 716 | 49 | +1.9 |
|  | 555 | gi\|42415475 | Protein disulfide-isomerase precursor | *P4hb/Pdia1* | 57.4 | 4.8 | 760 | 45 | +1.6 |
|  | 621 | gi\|568975124 | PREDICTED: heterogeneous nuclear ribonucleoprotein H isoform X8 | *Hnrph1* | 47.9 | 6.1 | 956 | 46 | +1.9 |
|  | 624 | gi\|148677504 | ATP synthase, H+ transporting, mitochondrial F1 complex, alpha subunit, isoform 1, isoform CRA_h | *Atp5a1* | 54.9 | 9.4 | 150 | 14 | +1.9 |
|  | 669 | gi\|70794816 | Enolase 1B, retrotransposed | *Eno1* | 47.5 | 6.4 | 586 | 62 | - 1.8 |
|  | 693 | gi\|114205428 | Reticulabin-2 isoform 1 precursor | *Rcn2* | 37.3 | 4.3 | 251 | 38 | +2.4 |
|  | 751 | gi\|4501885 | Actin, cytoplasmatic 1 | *Actb* | 42.1 | 5.3 | 513 | 45 | - 1.7 |
|  | 808 | gi\|157909797 | Mitochondrial import receptor subunit TOM40 homolog | *Tomm40* | 38.3 | 7.6 | 602 | 58 | - 1.9 |
|  | 851 | gi\|148694498 | mCG49244 | *C9orf156* | 21.8 | 5.7 | 745 | 76 | +2.6 |
|  | 963 | gi\|6679937 | Glyceraldehyde-3-phosphate dehydrogenase isoform 2 | *Gapdh* | 36.1 | 8.4 | 174 | 15 | - 2.0 |
|  | 1041 | gi\|6755965 | Voltage-dependent anion-selective channel protein 2 | *Vdac2* | 32.3 | 7.4 | 829 | 65 | - 1.6 |
|  | 1050 | gi\|743485 | YL2 protein | *C1qbp* | 23.8 | 4.4 | 91 | 13 | - 2.6 |
|  | 1134 | gi\|114326546 | Phosphoglycerate mutase 1 | *Pgam1* | 28.9 | 6.7 | 683 | 77 | +1.8 |
|  | 1201 | gi\|158937312 | Heat shock protein beta-1 | *Hspb1* | 23.1 | 6.1 | 77 | 26 | - 11.0 |
|  | 1209 | gi\|160298217 | Glutathione S-transferase A4 | *Gsta4* | 25.6 | 6.8 | 469 | 49 | - 3.4 |
|  | 1450 | gi\|148677406 | Cytochrome b-5, isoform CRA_b | *Cyb5b* | 13.3 | 5.7 | 122 | 41 | - 1.9 |
|  | 1545 | gi\|56709486 | Fatty acid binding protein 7, brain | *Fabp7* | 15.2 | 5.1 | 542 | 87 | +7.6 |
|  | 1557 | gi\|999883 | Chain A, crystal structure of cellular retinoid-acid-binding proteins I and II in complex with all-trans retinoid | *CrabpI* | 15.6 | 5.3 | 928 | 92 | +13.7 |
| **Set #2 (SwissProt)** | 268; 272; 276 | Q8K2B3 | Succinate dehydrogenase [ubiquinone] flavoprotein subunit, mitochondrial | *Sdha* | 73.6 | 7.3 | 546; 582; 320 | 14; 16; 11 | - 2.1; - 2.3; - 3.0 |
|  | 278 | Q8CG48 | Structural maintenance of chromosomes protein 2 | *Smc2* | 13.5 | 9.1 | 56 | 19 | +4.4 |
|  | 279 | P42932 | T-complex protein 1 subunit theta | *Cct8* | 60.1 | 5.3 | 56 | 31 | +4.7 |
|  | 316 | P56480 | ATP synthase subunit beta, mitochondrial | *Atpb* | 56.3 | 5.1 | 402 | 17 | +6.3 |
|  | 522 | Q8BFR5 | Elongation factor Tu, mitochondrial | *Tufm* | 49.9 | 7.9 | 356 | 14 | - 2.3 |
|  | 938 | P97823 | Acyl-protein thioesterase 1 | *Lypla1* | 25.0 | 6.2 | 105 | 10 | +2.0 |

SC = sequence coverage; FC = fold change.
