# Supplementary figures and images for: Membrane-enriched proteome changes and prion protein expression during neural differentiation and in neuroblastoma cells
Source: BMC Genomics. 2017 Apr 22;18:319. doi: 10.1186/s12864-017-3694-6 (PMC5401558; doi:10.1186/s12864-017-3694-6)

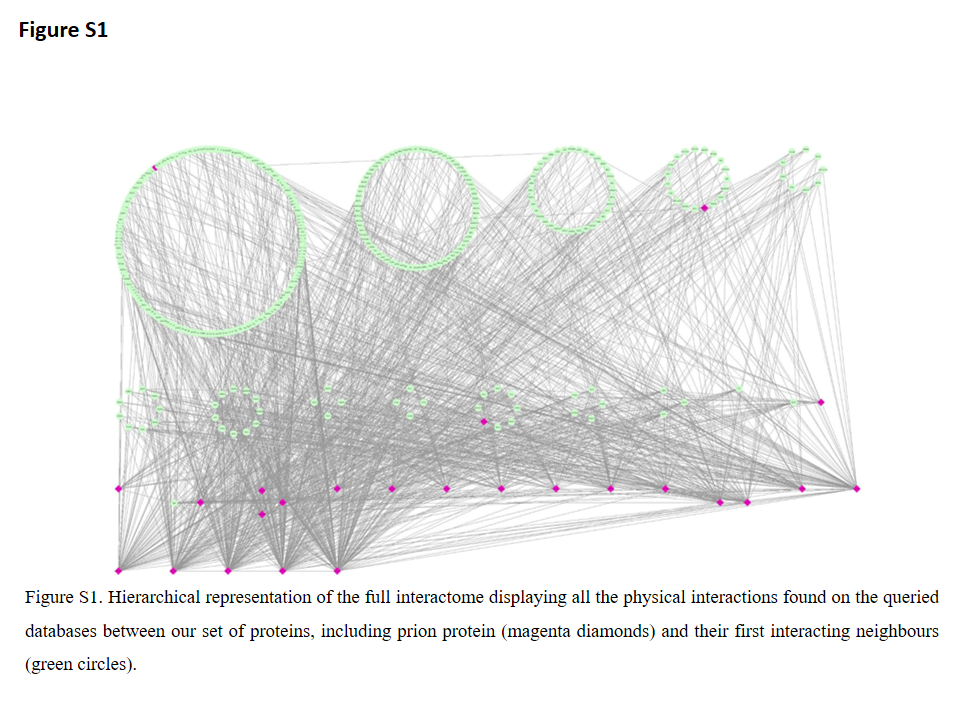

Supplement: Supplementary file 2 — Hierarchical representation of the full interactome displaying all the physical interactions found on the queried databases between our first set of differentially abundant proteins, including prion protein (magenta diamonds) and their first interacting neighbours (green circles). (TIF 569 kb) [file 12864_2017_3694_MOESM2_ESM.tif]

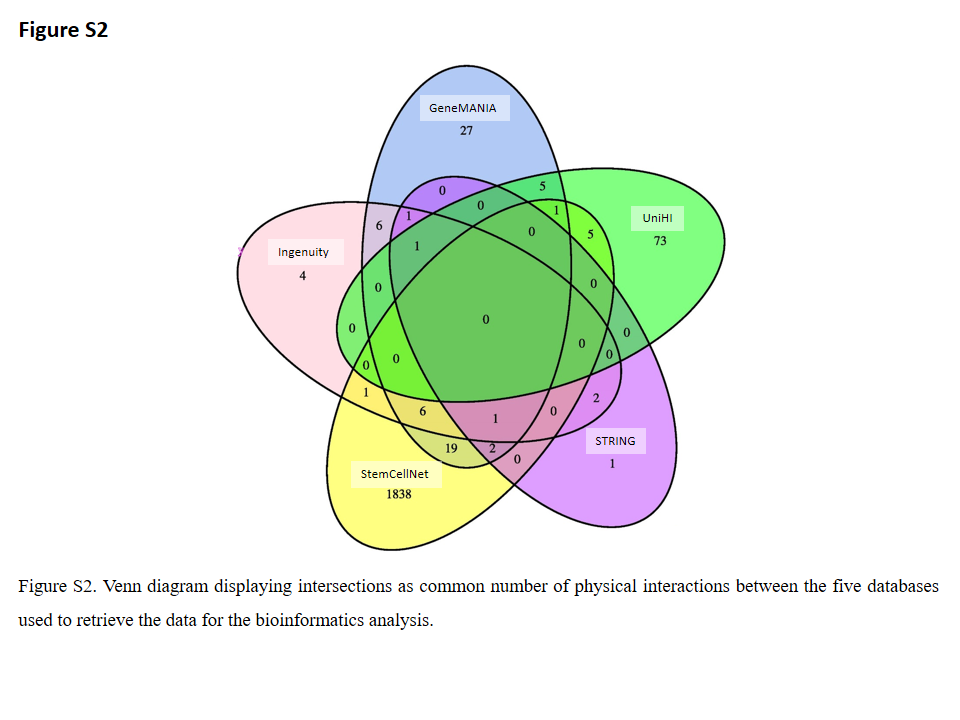

Supplement: Supplementary file 3 — Venn diagram displaying intersections as common number of physical interactions between the five databases used to retrieve the data for the bioinformatics analysis. (TIF 240 kb) [file 12864_2017_3694_MOESM3_ESM.tif]
